# Reactive Searching and Infotaxis in Odor Source Localization

**DOI:** 10.1371/journal.pcbi.1003861

**Published:** 2014-10-16

**Authors:** Nicole Voges, Antoine Chaffiol, Philippe Lucas, Dominique Martinez

**Affiliations:** 1 CNRS, LORIA, UMR 7503, Vandoeuvre-les-Nancy, France; 2 Inserm, UMR S968, Institut de la Vision, Paris, France; 3 INRA, UMR 1392, Institute of Ecology and Environmental Sciences of Paris, Versailles, France; Northeastern University, United States of America

## Abstract

Male moths aiming to locate pheromone-releasing females rely on stimulus-adapted search maneuvers complicated by a discontinuous distribution of pheromone patches. They alternate sequences of upwind surge when perceiving the pheromone and cross- or downwind casting when the odor is lost. We compare four search strategies: three reactive versus one cognitive. The former consist of pre-programmed movement sequences triggered by pheromone detections while the latter uses Bayesian inference to build spatial probability maps. Based on the analysis of triphasic responses of antennal lobe neurons (On, inhibition, Off), we propose three reactive strategies. One combines upwind surge (representing the On response to a pheromone detection) and spiral casting, only. The other two additionally include crosswind (zigzag) casting representing the Off phase. As cognitive strategy we use the infotaxis algorithm which was developed for searching in a turbulent medium. Detection events in the electroantennogram of a moth attached to a robot indirectly control this cyborg, depending on the strategy in use. The recorded trajectories are analyzed with regard to success rates, efficiency, and other features. In addition, we qualitatively compare our robotic trajectories to behavioral search paths. Reactive searching is more efficient (yielding shorter trajectories) for higher pheromone doses whereas cognitive searching works better for lower doses. With respect to our experimental conditions (2 m from starting position to pheromone source), reactive searching with crosswind zigzag yields the shortest trajectories (for comparable success rates). Assuming that the neuronal Off response represents a short-term memory, zigzagging is an efficient movement to relocate a recently lost pheromone plume. Accordingly, such reactive strategies offer an interesting alternative to complex cognitive searching.

## Author Summary

The moth mating race is a suitable model case for studying the efficiency of various search strategies and to compare them to real-world behavior. All there is to guide olfactory navigation are simple sporadic clues, i.e., single pheromone detections. Thus, a pheromone seeking male relies on a specifically adapted behavior where action selection is triggered by simple perceptional events. They switch between stereotypical movement sequences, as, for example, upwind surge and crosswind casting. This behavior can be either a consequence of cognitive processing or a reactive reflex of fixed action patterns. Suggesting a direct relationship between neuronal central activity and such action patterns, we combine and implement them as reactive strategies. We also employ infotaxis, an artificial intelligence algorithm specifically developed for searching in turbulent odor plumes. Using these strategies in cyborg experiments, we obtain and compare the resulting search trajectories. Our results indicate that complex, computationally expensive search strategies like infotaxis are not necessarily better than simple reactive ones. With respect to our set-up, reactive searching yields the shortest trajectories if and only if it includes a crosswind zigzagging phase that represents a short-term memory. Thus, already a minimal bit of simplistic memory can produce very efficient goal-directed behavior.

## Introduction

The efficiency of male moths searching for females is astonishing. In spite of serious difficulties, as, for example, large distances, sharp time constraints, and sparse discontinuous clues, their olfactory pheromone system usually guarantees a successful encounter [1], [2]. Far from the pheromone emitting female, odor plumes consist of sparsely distributed pheromone patches [3], leading to rare, intermittent detections [4]–[6].

This mating race is not only fascinating in itself but also particularly convenient to study the chain linking perception to action, and for the investigation of search tasks in general. The advantages are the rich adaptive behavioral repertoire of insects generated by a relatively simple neuronal system [7], [8], a clear instinct-based task, and its suitability for testing and comparing different types of search strategies. Moths, as well as other insects, have developed a specifically adapted behavior, in addition to a specialized neuronal subsystem for the processing of pheromone information [9], [10]. Experimental evidence indicates a two-step behavioral strategy [1], [5], [11]–[13]: sensing a pheromone patch induces an upwind surge [5], towards the pheromone emitting source (the female). Upon loosing the scent, they switch to crosswind (zigzag) casting [11], [12], [14]–[16], or looping or spiraling [14], [15], [17], [18]. Spiraling is typically done by walking insects.

An important factor is the olfactory stimulus [4], [5], [14], e.g., the pheromone dose or the pulsation frequency. The latter relates to another important factor, the presence of an air flow in odor-modulated anemotaxis. Inspired by the observations detailed above, various models of *reactive search strategies* have been suggested and modified [2], [19]–[21]. They are based on predefined movement sequences which are typically triggered by odor perceptions. Such biologically inspired strategies can be employed to locate pheromone sources [22] or other odors [23]–[26], given appropriate sensors. In general, bio-inspired methods are widely discussed to overcome the challenges in chemical sensing [26], [27].

A powerful alternative to reactive searching is the more sophisticated and computationally rather expensive approach of using *cognitive strategies*, e.g. [6], [28], [29] with respect to searching without continuous (or smooth) chemical gradients. Such methods produce an adaptive behavior as current perceptions are weighted by past clues and actions, i.e., learning and memory are typically involved. The infotaxis strategy [6] is based on Bayesian inference to maximize the information gain about the location of the source in a turbulent medium. Originally implemented as a simulation, infotaxis can be used in a real-world set-up in combination with a robot in order to track a thermal source [29], [30].

Such experimental tests of theoretical models are particularly important for applied research as they point out real-world issues and limitations that mere simulations cannot account for. This is true for both studies involving reactive [8], [26] and cognitive strategies [31], [32], as well as for gradient based underwater chemo-orientation using a biomimetic robot lobster [33]. Another example for such studies is biologically-inspired chemical plume tracing using an autonomous underwater vehicle [23], [24], [34]. Moreover, the interplay between robotics and insects offers the possibility to investigate the insect's behavior [8], [35]–[37] while specifically modifying the experimental conditions.

**Figure 1:**
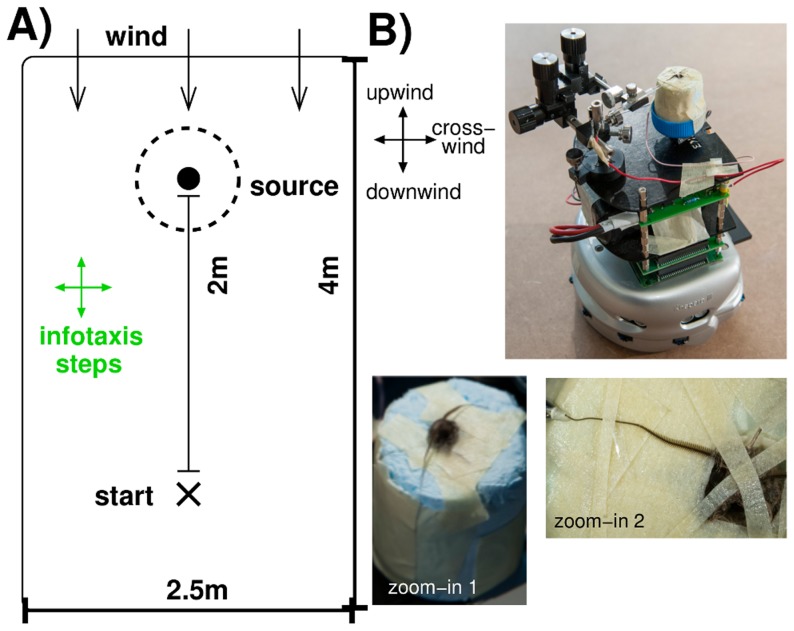
Experimental set-up of the cyborg's search task. (A) Schematic general set-up: the cyborg starts 2 m from the pheromone source in a 2.5
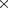
4 m region. A fan provides a wind blowing from the top (towards the cyborg). (B) Photo of our cyborg: a Khepera III robot with a moth fixed in a styrofoam roll. Zoom-in 1: top of the styrofoam roll with the insect's head and the two antennae on the outside. Zoom-in 2: one antenna enters the tip of a glass electrode. Photographs by H. Raguet — INRIA.

**Figure 2:**
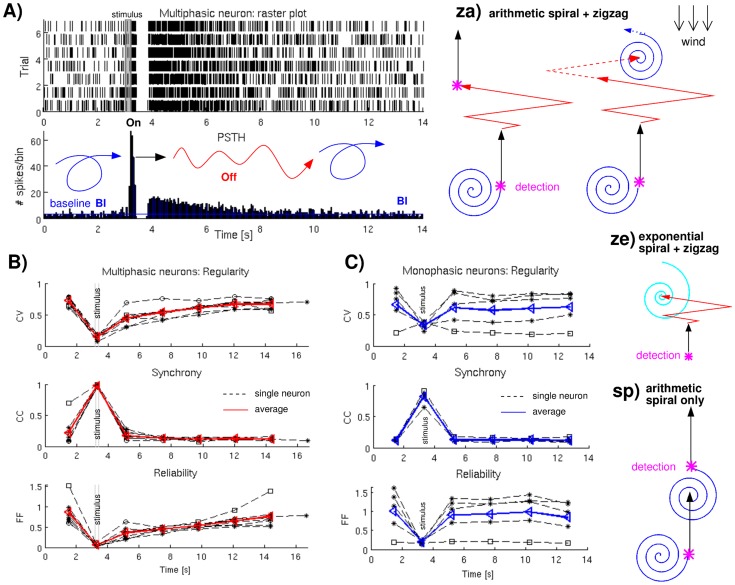
Reactive search strategies and their biological motivation. (A) MGC recordings for pheromone stimulation: spike times for seven trials of one neuron and the corresponding average firing rate over time (Peri-Stimulus-Time-Histogram): inhibition separates the On from the Off response which smoothly decreases to baseline firing (Bl). (B) Analysis of MGC recordings of multiphasic neurons: Calculating the regularity (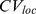) and reliability (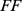) over time exhibits an Off phase, whereas Off and baseline firing show uniformly low synchrony values (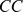). Dotted black lines represent single neuron trials, the red line gives the averages. (C) Analysis of MGC recordings of monophasic neurons: neither synchrony nor regularity nor reliability over time exhibit any Off phase. Dotted black lines represent single neuron trials, the blue line gives the averages. (Right side: za, ze, sp) Schematic representation of the corresponding movement sequences: Bl 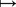 spiraling, On 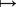 upwind surge, and Off 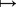 zigzagging (if considered) which are combined into three search strategies, *sp*, *za*, and *ze*.

Using such an approach, we compare the strategies detailed above: reactive versus cognitive searching in dependence of the stimulation strength for a turbulent air flow. Our cyborg, a male moth *Agrotis ipsilon* mounted on a mobile robot [22], has to find the pheromone source located two meters upwind from the starting position, see Fig. 1. The turbulent air flow yields sparsely distributed pheromone patches. Their detections, recorded from the antenna, control the robotic movements via three biologically motivated reactive search strategies and one cognitive infotaxis strategy [6]. The reactive strategies are derived from an analysis of electrophysiological recordings from the macroglomerular complex (MGC), the first cerebral relay for the processing of pheromone information perceived by olfactory receptor neurons (ORNs) in the moths' antennal lobe [9], [38]. They show triphasic neuronal responses (On, inhibition, Off) to pheromone stimulation [22], [39]. The origin of the inhibitory phase was the focus of [22] who employed reactive searching implemented on a cyborg in order to provide evidence that reactive searching could be mediated by multiphasic responses. We here provide a detailed comparison of cognitive versus reactive searching, in dependence of the stimulus dose, focusing on the analysis of the resulting trajectories. Based on the occurrence of multiphasic responses, we consider the strategies detailed in Fig. 2: (*sp*) combines surge and arithmetic spiral casting, (*za*) and (*ze*) combine surge and a two-step casting sequence composed of crosswind casting, i.e., zigzagging followed by arithmetic or exponential spiraling, respectively. The single movement sequences are motivated by biological findings on behavioral insect data: straight upwind surge [2], [5], zigzagging with an increasing step size [2], [40], and spiral casting [15], [18]. In addition, we contrast our search trajectories to those resulting from behavioral experiments where a walking silkmoth tracks a pheromone source in a wind tunnel [36].

We consider in this article the following questions: is complex cognitive searching superior to using simple reactive strategies? What is the influence of the stimulus strength? How can the resulting search trajectories be characterized and compared? Do they show common features and how do they relate to behavioral data? We expected a clear and overall predominance of infotaxis since the algorithm involves memory and learning, and it has been proven to be very successful in computer simulations [6], [41], [42]. We found, however, that reactive searching can be more efficient if it includes a response reminiscent of casting.

We first present the biological motivation of the implemented reactive search strategies. We then detail the results of using these strategies, as well as the infotaxis algorithm, in cyborg experiments. On the one hand, we focus on basic features as success rate and efficiency, expressed by the corresponding trajectory lengths. On the other hand, we also aim at a more detailed characterization of these search paths, including a qualitative comparison to behavioral data [36]. Finally, we discuss our findings, in particular in terms of which approach is more efficient, under which circumstances, and why.

## Results

We first detail the analysis of the neuronal MGC recordings. Subsequently, we relate the different regimes therein to the reactive search strategies used in the cyborg experiments. After a brief introduction of the infotaxis strategy, we present the results of comparing various search strategies in cyborg experiments. Finally, we relate and compare patterns occurring in our cyborg trajectories to those of walking moths published in [36].

### Electrophysiological results

We consider the electrophysiological MGC recordings of 8 multiphasic neurons and 6 monophasic neurons (Fig. 2, in total 58 and 53 single trials, respectively). The Off phase in multiphasic neuronal responses to pheromone stimulation in the MGC is apparent in the firing rate [9], [22], [39]. We find that it is also apparent in the firing regularity and firing reliability which are characterized by the coefficient of variation and the Fano Factor, respectively. Fig. 2A shows the spike times of seven trials of a multiphasic MGC neuron. Upon pheromone stimulation, firing abruptly increases from the baseline (Bl) and produces an On response, followed by an inhibitory phase. After inhibition, neuronal firing restarts at a higher rate than Bl activity which then slowly decays back to baseline. We call this transient intermediate phase the Off response. The Off is apparent in the Peri-Stimulus-Time-Histogram (PSTH, the average firing rate over time), in terms of spiking regularity and reliability, as shown in Fig. 2B. Both the coefficient of variation and the Fano Factor are close to one during irregular and unreliable Bl firing, drop to approximately zero during the On, and show intermediate values during the Off while gradually reapproaching one. In contrast, the correlation coefficient, representing spike time precision (Methods, part 1), exhibits no Off phase. Both the increase in synchrony, i.e. spike time precision, from baseline (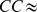
0) to On (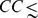
1) and the decrease back to zero are abrupt.

For comparison, we also analyze monophasic MGC responses to pheromone stimulation, shown in Fig. 2C. Such neurons also respond with an On, i.e., an abrupt increase in firing (data not shown, see [22, Fig.2]), similar to the On response of multiphasic neurons: there 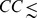
1, 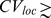
0, 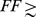
0). Yet, for monophasic neurons the increased spiking during the On switches immediately back to baseline spiking, there is neither an inhibitory, nor an Off phase. Both firing regularity and reliability switch directly back to their baseline values: 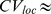
0.7 and 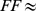
1, respectively.

### Reactive search strategies

On the right side of Fig. 2 we present the reactive search strategies associated with the different in the neuronal MGC responses to pheromone stimulation. Note that the wind is assumed to blow from the top and that mean wind direction and speed are fixed. Basically, we distinguish between two assumptions: the *sp* strategy neglects the Off while the other two strategies (*za* and *ze*) comprise a zigzag casting sequence representing the Off in our multiphasic neurons (see Methods, part 2). The *sp* strategy could thus model the behavior based on the activity of monophasic neurons.

As initial movement, we choose arithmetic or exponential spiraling which represents baseline firing. We assume that each detection event initiates a straight upwind surge representing the On. If there is no subsequent detection, the movement changes either into Off zigzagging followed by baseline spiraling, or it directly switches back to baseline spiraling (*sp*, using arithmetic spirals). Note that the cyborg stops zigzagging after a fixed period of 19 s (if there is no further detection): as we record from the antenna and not from the MGC, we do not have access to the length of the Off. Our *za* and *ze* strategies combine zigzagging with either arithmetic or exponential spirals [43] in order to test whether exponential spiraling yields an increases in efficiency [22, Suppl. Information].

We do not consider reactive searching without spiral movements as we expect relatively high failure rates due to the missing downwind component [25]. Consequently, the agent cannot reorient appropriately after passing (and missing) the source. This happens, for example, whenever detections occur close to, but downwind and laterally shifted with respect to the source position.

### Cognitive searching with infotaxis

We now briefly introduce the infotaxis algorithm [6]. It uses Bayesian inference to localize the source of an odor plume in a turbulent medium. It combines two mechanisms: *exploitation* (i.e., approaching the source based on perceived information) and *exploration* (i.e., maximizing the information gain). The exploration mode predominates if there is nearly no information available. Long periods of time with no odor encounter broaden the posterior distribution and compel the agent to explore the environment in large patterns. On the contrary, if many detections indicate that the source is close, the exploitation mode triggers more localized movements. Starting from a prior probability distribution for the location of the source, the agent accumulates information while exploring its environment. Both odor detections and non-detections contribute to the ongoing update of the estimated spatial probability distribution. Infotaxis has been shown to function very well in computer simulations (see Introduction): typical failure rates are close to zero, even for ‘no wind’ or ‘no stimulus’ conditions (the agent continues to search until the source is found). Its movements are discretized steps and it allows only for four movement directions. For each step, the direction is determined as that which maximizes the entropy reduction in the probability distribution of the source location. More details are given in Methods, part 3.

### Comparison of different strategies

We now compare the results of our cyborg experiments, i.e., search trajectories obtained for three reactive search strategies and infotaxis stimulated with different pheromone concentrations (no pheromone, minimum, medium, and maximum concentration, see Methods, part 2). The number of successful and failure trials are given in Table 1.

**Table 1:** Experimental robotic trials.

Successful/total trials	*sp*	*za*	*ze*	*it*
no pheromone	5/27	3/18	4/29	21/29
minimum 0.1	32/43	34/38	38/48	21/22
medium 0.3	65/71	65/73	36/42	24/25
maximum 1	43/49	66/74	49/56	21/24

List of robotic experiments considered for the statistical analysis of the success rate. The trajectory analysis includes only the successful trials. Each entry S/T indicates the number of successful trials S out of the total number of trials T. Spiraling only (*sp*), arithmetic spiral & zigzagging (*za*), exponential spiral & zigzagging (*ze*) are the trials based on reactive search strategies, *it* trials are obtained using cognitive infotaxis searching. For a definition of successful or failure trial see Methods, part 3. The large variations in the number of trials are due to the need to exclude trials, as well as differences in the technical requirements.

#### Success rates

**Figure 3:**
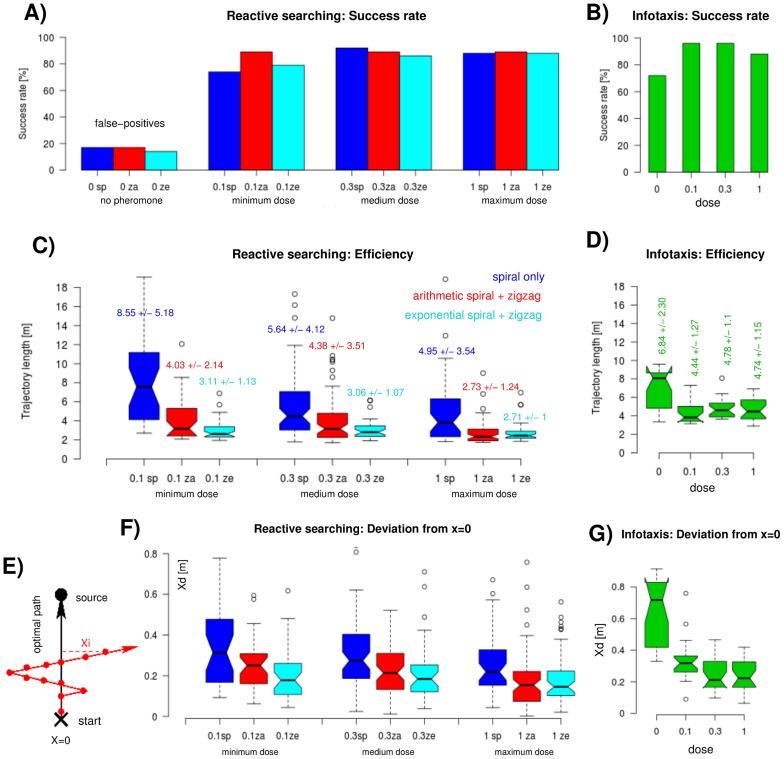
Success rates, trajectory lengths, and deviation from the optimal path. (A) Success rates of reactive search strategies, different colors indicate different strategies (legend in C), grouping indicates different stimulation doses (three doses plus no stimulation). (B) Success rates of cognitive searching with infotaxis (three doses plus no stimulation). (C) Trajectory lengths of reactive search strategies, different colors indicate different strategies, grouping indicates different stimulation doses. (D) Trajectory lengths of cognitive searching with infotaxis (three doses plus no stimulation). (E) Schematic drawing to explain the 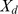 measure: the average of horizontal deviations (Xi) from trajectory to shortest path between start and source. (F) Deviation from the optimal path (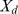) for reactive searching, different colors indicate different reactive strategies for the three groups of pheromone doses. (G) Deviation from the optimal path (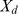) for cognitive searching (three doses plus no stimulation). Box plots are explained in the Methods, part 2, the numbers indicate mean 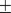 standard deviation.

The success rates, i.e., the percentage of trials in which the cyborg locates the source, are very similar for the three reactive strategies, see Fig. 3A: 89% (*sp*), 85% (*za*), and 84% (*ze*), averaged across different doses. Cognitive searching yields a sightly higher success rate of 93% (Fig. 3B), averaged over all stimulated trials (or 88% if the ‘no stimulus’ condition is included). We find that there is no dependency on the stimulus dose: 85% (minimum dose), 91% (medium dose) and 88% (maximum dose) successful trials, averaged across all strategies. Only the differences between reactive trials with and without stimulation are pronounced. There are up to 17% successful reactive trials without stimulation due to occasional false detections, i.e. false positive trials. The success rate for infotaxis (*it*) trials without stimulation is 73%. These are not called false positives, since infotaxis is supposed to be successful even without stimulation (see above and Methods, part 3). Compared to 93% successful trials with stimulation, 73% is rather low. The reason is that such trajectories often run close to the boundaries of the search space (the experimental field) so that even minor odometry errors (Methods, part 2) potentially cause the cyborg to leave the search space. Thus, reactive searching fails if there is not enough information (e.g., no pheromone source) whereas infotaxis is still able to reach the source (even for the ‘no pheromone’ condition) by exploring the environment in an efficient way.

#### Efficiency

We compare the efficiency of different search strategies in dependence of the pheromone dose by means of the resulting trajectory lengths, shown in Fig. 3C and 3D. Note that there is a minimum length for all search trajectories, namely, the shortest possible straight-line distance between start and source. Some of our trajectories are indeed up against this boundary. First, reactive searching with zigzag movements representing the Off is clearly more efficient than spiraling, only. For all doses, *ze* yields the shortest search paths, closely followed by *za*, while *sp* trajectories are clearly the longest ones. Second, cognitive searching using infotaxis produces shorter trajectories than using the *sp* strategy but cognitive paths are longer than those including Off zigzagging. Third, the higher the pheromone dose, the shorter the reactive trajectories. In contrast, the lengths of cognitive *it* trajectories obtained with pheromone stimulation differ only slightly and the minimum dose produces the shortest paths.

A global ANOVA yields a statistically significant difference for both strategy and dose (p
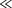
0.001), and a weak interdependency (p = 0.06) between these two factors. Single pairwise comparisons, however, exhibit a more diverse pattern: the length differences between *za* and *ze* versus *sp* are statistically significant, (p
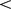
0.001 up to p
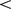
0.05), whereas this is not the case for *za* versus *ze* trajectories. The length differences between trajectories resulting from reactive and cognitive searching are mostly significant: *sp* versus *it* for the minimum dose (p
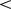
0.01), *za* versus *it* in case of medium (p
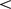
0.01) and maximum (p
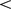
0.001) dose, and all *ze* versus *it* pairs (p
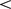
0.001). Pairwise comparisons between different doses (for each single strategy) yield a more variable pattern: p
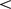
0.001 for minimum vs. maximum dose in case of *za* and *sp* trajectories, p
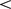
0.001 for medium vs. maximum (minimum) dose in case of *za* (*sp*, p
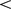
0.01) and for all *it* pairs including the ‘no pheromone’ condition (in contrast to p
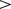
0.05 for all *it* pairs with stimulation). More details are given in Table 2. The difference between *ze* path lengths obtained for varying doses, as well as between *sp*, *za* and *ze* path lengths obtained for the maximum dose are particularly small because these trajectories are close to the minimum length.

**Table 2:** Statistics of pairwise comparisons.

strategy	dose	path length	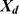	# turns	# detections
*sp*,*za*	min	***		***	**
*sp*,*za*	med	*	**	***	*
*sp*,*za*	max	***	**	*	
*sp*,*ze*	min	***	***	***	
*sp*,*ze*	med	***	**	***	
*sp*,*ze*	max	***	**	***	
*za*,*ze*	min		*		
*za*,*ze*	med				
*az*,*ze*	max			*	
*it*,*sp*	min	**		***	
*it*,*sp*	med			***	***
*it*,*sp*	max			***	***
*it*,*za*	min		*	***	
*it*,*za*	med	**		***	***
*it*,*za*	max	***	*	***	***
*it*,*ze*	min	***	***	***	
*it*,*ze*	med	***		***	***
*it*,*ze*	max	***	*	***	***
*za*	min,med				
*za*	min,max	***	**	***	**
*za*	med,max	***	**	**	*
*sp*	min,med	**			
*sp*	min,max	***	**	**	
*sp*	med,max				
*ze*	min,med				
*ze*	min,max	*		*	
*ze*	med,max	*			
*it*	min,med		**	***	***
*it*	min,max		**	***	***
*it*	med,max				
*it*	min,no	***	***	*	***
*it*	med,no	**	***	***	***
*it*	max,no	**	***	***	***

Detailed list of the results of the pairwise comparisons required for the statistical analysis. Compared are the differences in the search path length, the deviation from the optimal path 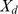, the total number of turns and detections with respect to four search strategies (spiraling only *sp*, arithmetic spiral & zigzagging *za*, exponential spiral & zigzagging *ze*, infotaxis *it*) and three pheromone doses (minimum, medium, maximum). For the *it* strategy we also include a ‘no pheromone’ condition. Each row refers to one pair: It indicates the p-value of the comparison between either two strategies for a given dose (upper part) or two doses for a given strategy (bottom part). *** indicates 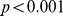, ** indicates 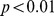, * indicates 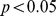, and no entry refers to 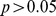.

**Figure 4:**
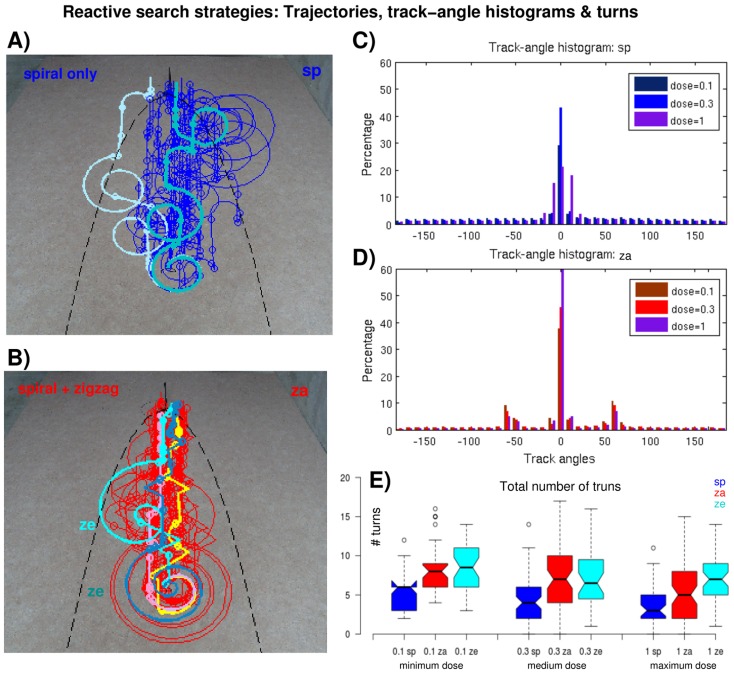
Reactive search trajectories. (A) Examples of *sp* search trajectories (spirals only, i.e., no Off), medium dose. For a better visualization single paths are plotted in distinct colors (cyan and light blue on top of mostly blue trajectories). The dots on the trajectories indicate pheromone detections. The black dashed line indicates the plume contour (see Methods). (B) Examples of search trajectories including Off zigzagging, medium dose. Red, yellow and pink trajectories use arithmetic spirals (*za*), bluish trajectories originate from assuming exponential spirals (*ze*). Identical conventions as in (A). (C and D) Track-angle histogram of *sp* and *za* trajectories, respectively, different colors indicate different pheromone doses. (E) Total number of turns for different stimulations, different colors indicate different reactive strategies for the three groups of pheromone doses, identical conventions as in Fig. 3.

Fig. 3F reveals that distinct lengths of reactive search trajectory are mainly due to a horizontal X-deviation from the optimal path (introduced in Fig. 4E, see Methods, part 3). This horizontal extent (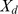) is larger for *sp* compared to *za* and *ze* trajectories and for lower versus higher pheromone doses. Pairwise comparisons show that the 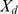 differences between the *sp* strategies and those including zigzagging are mostly significant (p
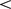
0.01), contrary to the differences between *za* and *ze*. With respect to different doses, 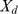 of *sp* and *za* trajectories differ significantly between minimum and maximum dose (p
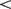
0.01) while this is not the case for *ze* trajectories. Hence, for reactive strategies, the horizontal deviation from the optimal path (Fig. 3F) coincides with dose- and strategy-induced differences in the path lengths (Fig. 3C). For cognitive trajectories, however, the relationship is more complex: again, the spatial spread 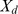 decreases with increasing pheromone dose as shown in Fig. 3G (p
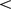
0.01 for pairs including the minimum dose, see Table 2). The shortest infotaxis trajectories, however, occur for the minimum instead of the maximum dose (Fig. 3D). Only the ‘no pheromone’ condition yields a pronounced horizontal deviation (p
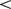
0.001 for all *it* pairs) together with particularly long trajectories.

#### Trajectories: Track-angles, turns and detections

**Figure 5:**
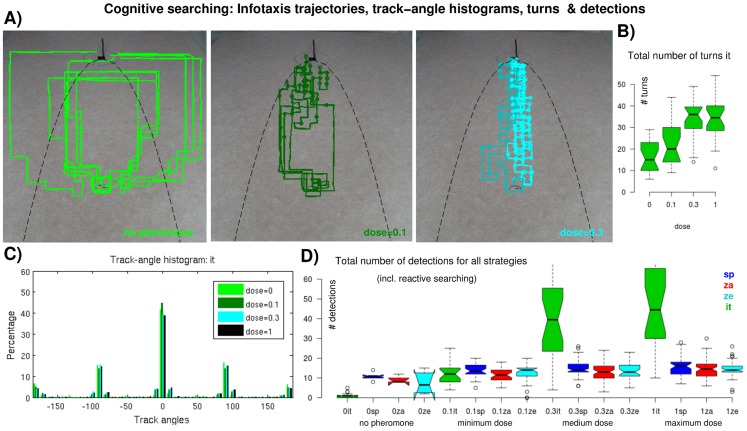
Cognitive search trajectories obtained using infotaxis. (A) Example *it* trajectories for no stimulation (green, left), minimum (dark green, middle) and medium (cyan, right) stimulation doses. The dots on the trajectories indicate pheromone detections. The black dashed line indicates the plume contour (see Methods). (B) Total number of turns in *it* trajectories for different stimulations. Identical conventions as in Fig. 3. (C) Track-angle histograms of *it* trajectories, different colors indicate different doses. (D) Total number of detections measured during reactive (*sp*, *za*, *ze*) and cognitive (*it*) searching using three stimulation doses and no stimulation. Identical conventions as in Fig. 3.

Fig. 4 shows some arbitrarily chosen trajectories obtained using reactive search strategies, the corresponding track-angle histograms, and a box plot on the total number of turns larger than 55°, in dependence of the pheromone dose. Similarly, Fig. 5 shows a selection of trajectories obtained using the cognitive infotaxis strategy, the corresponding track-angle histograms, and the corresponding number of turns (paths and track-angle histograms obtained with maximum stimulation are not shown, they resemble those obtained with medium stimulation).

Already a qualitative comparison of the reactive trajectories shows that two-phase casting including Off zigzagging leads to more efficient trajectories than spiral casting, only (Fig. 4A and 4B). Spiral movements contribute equally to all track-angle bins, whereas upwind surge theoretically only increments the zero bin. In practice, surge also contributes to neighboring bins (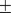
10°, Fig. 4C) because of imprecise robotic movements (Methods, part 3). The same argument applies for the peaks in other track-angle histograms (Fig. 4D and 5C). The *sp* histogram shows one single peak representing the upwind surge associated with the On response while the *za* histogram exhibits two additional peaks at approximately 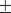
60° due to Off zigzagging. The peaks principally reflect experimentally observed behavior: Upwind movements after pheromone detections yield one central peak while crosswind casting after odor loss yields a symmetric two peak distribution [13], [15], [40]. The remaining bars (outside the peaks) in *za* and *ze* histograms (Fig. 4D) are smaller than those of the *sp* histogram (Fig. 4C) since spiraling occurs less, predominantly at the beginning (Fig. 4A). Regarding stimulation strengths, we see that higher pheromone doses leads to more surging, i.e., a higher central peak in both histograms. The opposite is true for the 60° peaks in the *za* histograms: the higher the dose, the less zigzagging. The number of turns in Fig. 4E is lowest in *sp* trajectories while *za* and *ze* trajectories naturally contain significantly more turns (mostly p
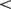
0.001 for *sp* versus *za* and *ze*, see Table 2).

A qualitative inspection of the samples in Fig. 5A indicates that cognitive trajectories noticeable depend on the pheromone dose. Yet, the track-angle distributions of *it* trajectories (Fig. 5C) are not distinguishable. They simply reflect the fact that infotaxis permits only four movement directions: a (fixed length) step forward (0°), backward (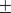
180°), left or right (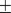
90°). These steps are concatenated in distinct ways, depending on the pheromone dose (Fig. 5A). Fig. 5B provides an explanation: the number of turns in infotaxis trajectories is positively correlated with the stimulation dose, i.e., medium and maximum stimulation yield particularly curvaceous paths (p
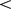
0.001 for *it* minimum or medium vs. maximum dose). In terms of reactive trajectories, however, the number of turns slightly decreases with increasing dose (partially significant, see Fig. 4E and Table 2). Instead of simply moving straight forward to the source, the cyborg (under infotaxis control) turns a step to the left (or right) and back while principally moving upwind (Fig. 5A, right).

Fig. 5D indicates that number of pheromone detections is exceptionally high for *it* trajectories stimulated with medium or maximum dose (p
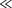
0.001 for all corresponding comparisons, see Table 2). As expected, there are generally more detections for higher pheromone doses, as well as more detections for zigzagging than for spiral trajectories. Both effects are partially significant (p
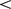
0.01 for minimum vs. maximum dose in *za* paths, and for *za* vs. *sp* at minimum dose, see Table 2). Thus, the length of reactive trajectories is predominantly determined by their horizontal spread: a higher pheromone dose leads to more detections which yields more surging and less turning which results in shorter paths. Concerning infotaxis one needs to distinguish between the following two cases: In the ‘no pheromone’ condition *it* paths are particularly long due to a large horizontal spread (combined with few turns). Otherwise, in case of stimulated trials, the path length increases with the pheromone doses due to the high number of turns which are related to the exceptionally high number of detections (see subsection Infotaxis) — while the horizontal spread decreases.

One might wonder about the low success rate for reactive trials with no stimulation (Fig. 3A) as the corresponding number of detections is nearly as high as for the minimum dose (cf. Fig. 5D). Such false detections occur randomly, i.e., they are typically not related to the relative positioning of cyborg and source and thus mostly misleading, causing the cyborg to miss the source. Nevertheless, some false detections accidentally guide the cyborg towards the source, leading to 17% false positive trials. Hence, Fig. 5D confirms that reactive strategies fail if there are not enough or misleading detections whereas this is not the case for infotaxis (cf. Fig. 3A and 3B).

### Qualitative comparisons with moth trajectories

**Figure 6:**
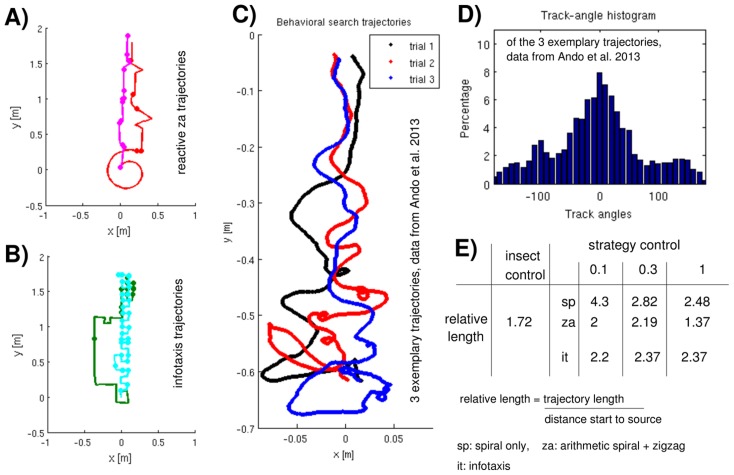
Contrasting juxtaposition of our trajectories to behavioral data. (A) Two representative reactive (*za*) search trajectories: the pink path is characteristic for a maximum stimulation dose, the red path is a typical result of medium or low stimulation. The dots on the trajectories indicate pheromone detections. (B) Two representative infotaxis trajectories: the cyan path is characteristic for using the maximum or medium dose, the green path is a typical result of minimal stimulation. (C) Three exemplary behavioral search trajectories, provided by the Kanzaki-Takahashi Laboratory (University of Tokyo) [36]. A silkmoth walking in a wind channel started at (0,0.6). The source was at (0,0) but was considered to be reached when within 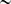
5 cm of the latter. (D) Track-angle histograms (bin size  = 10°) of the trajectories shown in (C). (E) Tabular comparison of the resulting trajectory lengths of strategic versus behavioral searching.

With Fig. 6 we aim at a qualitative comparison between our strategy driven trajectories and behavioral data provided by the Kanzaki-Takahashi Laboratory [36]. There, a silkmoth (*Bombyx mori*) walking in a wind tunnel tries to locate a pheromone source (relatively high pheromone concentration) located 60 cm away from the starting position [8], [36], [37]. Large parts of the trajectories are estimated to lay inside the odor plume. We show three arbitrarily selected behavioral search paths and the corresponding track-angle distribution. These are much broader than our histograms (Fig. 4C, 4D and 5C) but there is a central (surge) peak, as well as a second peak due to zigzagging, though here occurring at 100° and non-symmetric (probably due to the low number of samples). In order to permit a qualitative mapping, we selected two typical examples of reactive paths, as well as two representative examples of cognitive search paths, shown in Fig. 6A and 6B, respectively. Since the behavioral data (Fig. 6C) was obtained for a relatively strong stimulation, we here neglected cyborg data obtained for minimum or no stimulation, as well as particularly long or widespread paths. Behavioral trajectories exhibit no spirals but there are some circular sections, e.g., far from the source in trial 3, as well as some looping sequences (trial 2). The lack of spirals in behavioral compared to our reactive trajectories (Fig. 6A) could be due to the small distance between start and source (0.7 m) or due to cumulative navigational errors, similar to our odometry errors. For example, it has been suggested that the homing paths of desert ants are actually distorted spirals [18]. The insect-controlled trajectories in Fig. 6C are clearly dominated by zigzag patterns, but without increasing lateral amplitudes — as we assumed (Methods, part 2) for our *za* and *ze* strategies [2], [40]. Our reactive search paths contain many straight upwind movements representing surge sequences (Fig. 6A). Such isolated straight upwind sections also emerge in infotaxis trajectories, e.g., the dark green path in Fig. 6B, even though not as a consequence of detections but rather related to exploration movements. The behavioral trajectories, however, become less curvaceous towards the source; straightness occurs rather as a global instead of an isolated local feature (Fig. 6C). In terms of turning left and right while globally moving upwind, they resemble the cyan cognitive path in Fig. 6B) although horizontal movements in behavioral paths decrease towards the source. The latter feature, however, is also observable for the population of reactive *za* and *ze* trajectories (Fig. 4B).

## Discussion

After having substantiated the existence of an Off firing phase in multiphasic MGC responses to pheromone stimulation we defined two basic reactive strategies (Fig. 2): a simple one composed of surge and spiral casting (*sp*), and two more complex strategies including an additional Off zigzag phase following the surge sequence (*za*, *ze*). We applied these reactive strategies, as well as the cognitive infotaxis algorithm, in robotic experiments enabling our cyborg to locate a pheromone source. Reactive searching with Off zigzagging yielded the shortest trajectories, independent of the pheromone dose (Fig. 3). Infotaxis is less efficient but ensures slightly higher success rates, while reactive searching using only spiral casting was least efficient. The effect of the pheromone dose on success rates and path lengths was not as clear as expected. With respect to reactive strategies a higher dose led to shorter trajectories (Fig. 3C), to smaller deviations from the optimal path (Fig. 3F), and to more upwind surge (Fig. 4C and 4D). In terms of cognitive searching, however, the minimum dose yielded the shortest path — in spite of rather large deviations from the optimal path (Fig. 3D and 3G). Moreover, there was no effect of the dose on the track-angle histograms but the absolute number of turns increased with the dose (Fig. 5C and 5B).

Cognitive strategies are based on complex algorithms that involve memory and learning. Given the naive assumption of a linear relationship between costs in terms of complexity and profit, we expected infotaxis to be superior to reactive strategies — which is not what we found. We now take a closer look at some factors characterizing our search task: the distance between starting position and source, the strength of the wind and the pheromone dose. A higher dose induces more pheromone patches and thus augments the probability of pheromone detections (cf. Fig. 5D). Shorter distances to the source, as well as a stronger wind should have a similar effect. More frequent clues, in turn, should facilitate the search task. In general, infotaxis trials enabled many detections due to a slowly moving robot (Methods, part 2), i.e., a longer time span to perceive pheromone patches. This is particularly true at medium and maximum doses where the cyborg starts to detect very early and then advances by zigzagging along the centerline (Fig. 5A, right), generating further detections. Despite so many clues, the resulting trajectories are astonishingly long. We therefore suspect that our experimental set-up with only 20 steps between agent and source is not very appropriate for cognitive searching. It provides a rather simple task compared to the actual capabilities of infotaxis in computer simulations [6], [30], [41], [42] with more than 100 steps between agent and source. The basic difference between our real-word setup and infotaxis simulations is that we are confined to searching relatively close to the source because of experimental constraints whereas there are no such restrictions in computer simulations. Indeed, in more dilute conditions (lowest dose and ‘no pheromone’ condition), robot paths resemble typical infotactic trajectories previously observed in simulation.

With respect to our experimental conditions, reactive searching that includes Off zigzagging is obviously the optimal solution, in particular when combined with exponential spiraling. It has been already suggested that such a two-phasic casting yields shorter trajectories than spiraling only [22], but only for the maximum dose (cf. Introduction). Here, we demonstrate that this reduction is larger for more demanding tasks, and we investigate the dose dependency in terms of movement directions (Fig. 4). The explanation is as follows. The uncertainty about the location of the source is larger in the direction perpendicular to the wind than in the axis of the wind since the default search direction is upwind. Hence, zigzag movements perpendicular to the wind are more efficient than isotropic spirals as the potential information gain is larger. But what could be the origin of the Off in multiphasic MGC neuron firing? We hypothesize that it is an effect of the long tail of the pheromone response of ORNs [9], [38], [39]. A high number of ORNs connect to fewer (projection) neurons in the MGC [9], [10]. ORNs respond to a brief pheromone pulse with a rather steep increase in firing which then slowly decays back to baseline spiking [39]. The duration of this decay exceeds the end of the inhibitory phase of MGC neurons. Moreover, the slightly increased reliability during the Off (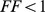) could also be explained by this hypothesis: there is still enough parallel ORN input to induce reliable spiking over different trials, but it is not enough synchrony to obtain precise spike timing (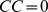).

Stimulus On and Off responses are typically reported for distinct neurons. A famous example is the On and Off cells in the visual system of vertebrates, e.g., in the cat's visual cortex [44], that enhance contrast information. The term “Off cell” typically refers to a neuron that primarily responds to a reduction in stimulation strength or to the end of a stimulation period. With respect to insect olfaction, separated On and Off ORNs in the cockroach antenna have been reported to encode opposite changes in the concentration of fruit odors[45], [46]. The Off response of certain neurons in the silkmoth's antennal lobe even contains information about the odor's identity [47]. Another example, related to behavioral switches, are two distinct neuronal populations in basal amygdala of mice that signal ‘fear on’ and ‘fear off’, respectively, initiating the appropriate behaviors [48]. In this article, however, On and Off originate from the same neuron, but emerge one after another. As usual, the On encodes the stimulus onset, i.e., a pheromone detection, while the Off signals that there has been no subsequent detection. If there are several subsequent detections (pulsation), there are also several subsequent (independent) On responses, each followed by an inhibitory phase [22], [39], [49]. Thus, in agreement with the conventions described above, our Off encodes the loss of a stimulus — while additionally carrying some timing information. We propose to consider Off zigzagging as a form of short-term memory. This concept is in good agreement with our hypothesis on its origin, the long-lasting ORN responses. Hence, the Off phase indicates that there has been a recent pheromone detection which has just been lost. In this case a behavioral switch to crosswind zigzagging is appropriate because the agent is probably inside the plume and just needs to locate the centerline [37], [50]. If, however, the scent has been lost a longer time ago (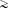
30s in our experiments), the corresponding movements should include a downwind component in case the agent already passed the source. We here assumed spiraling [22], [43] but looping [14], [15] or zigzagging with an angle 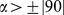
° [2], [13] would also be adequate. In this respect, reactive searching composed of surge and *two-phasic* casting establishes a kind of compromise strategy: predefined movements that include memory about the timing of the last detection. According to our results, such an approach seems to be the best choice for searching inside or close to the pheromone plume, i.e., if the agent is located downwind not too far from the source.

Obviously, our study cannot answer the question which search strategy moths actually use. However, we would like to stress that a moth using infotaxis would require to develop a cognitive map capturing information about all previous detections and their spatial positions with respect to the agent's locations. Instead, we provide some evidence on how efficient, adequate, and realistic various strategies are compared to behavioral data. Behavioral trajectories are shorter than all robotic paths — except for reactive zigzagging with maximum stimulation (Fig. 6E). Thus, under the given experimental conditions, insect behavior is generally more efficient than infotaxis and also than reactive searching. The problem with most biological data is that timing and positions of odor detections are not known. In any case, close to the source there are many detections. Then, infotaxis is particularly inefficient since it yields too many sharp turns, the trajectories being dissimilar to behavioral ones. The latter are not necessarily completely straight as assumed for our surge movements but exhibit a few turns of small curvature [36]. This is probably the reason for the superiority of zigzagging with maximum stimulation that yields very straight trajectories (pink in Fig. 6A). If there are only a very few detections, the exploration term of infotaxis yields rather straight upwind paths with some embedded loops (dark green in Fig. 6B). In contrast, behavioral paths show mostly zigzagging (with sharp turns and large lateral amplitudes), as well as some spiraling which is by far not as regular and long-lasting as assumed for our reactive strategies. Therefore, as alternative approach for reactive searching, we propose to prolong zigzagging and to introduce a dependency of both the lateral displacement and the turning angle on the recent number of perceived detections: the more recent detections the smaller the angle and the step size. Moreover, zigzag sequences are not only potentially related to multiphasic MGC responses, they have also been suggested to be linked to the so-called flip-flop activity of descending neurons in the silkworm [17], [51]. The big advantages of reactive searching are its proximity and adaptability to real-world biological data, as well as its simplicity in terms of computational requirements. Nevertheless, we speculate that cognitive searching, whether used in nature or not, is more appropriate if the agent's starting position is far outside the odor plume (dilute condition).

## Methods

### Electrophysiological recordings and their analysis

Neurons from the macroglomerular complex (MGC) were recorded from male *Agrotis ipsilon* Hufnagel during pheromone stimulation of the antennae. The pheromone stimulus is a blend of three components (ratio 4∶1∶4): *(Z)*-7-dodecenyl acetate, *(Z)*-9-tetradecenyl acetate and *(Z)*-11-hexadecenyl acetate. The stimulation lasted 200 ms. There are 4 to 10 trials for each neuron. Extracellular recordings were performed by inserting two glass electrodes filled with Tucson ringer into the MGC. After amplification the signal was band-pass filtered (0.3 to 5 kHz) and sampled at 16 kHz. Spike sorting (R-package SpikeOMatic) yielded single neuron signals. For more details see [38], [39].

We here analyzed the responses of 8 neurons that exhibited clear multiphasic responses (Fig. 2A and 2B): an excitatory On peak in the Peri-Stimulus-Time-Histogram (PSTH, the average firing rate over time), followed by an inhibitory phase and finally a more or less pronounced tonic excitatory Off phase [9], [22], [39]. For analysis, the recordings were subdivided into the following time intervals: baseline Bl, On, Off1, Off2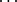 Off5 (Off6 for neuron 1). The separation between Bl and On onset was based on a segmentation algorithm described [22], Off1 starts directly after the inhibitory phase. The Off interval length (2.3 or 2.5 s) was chosen in a way that smooth changes during the Off are detectable and otherwise as large as possible. For comparison, we also investigated 6 monophasic neurons (separated in six time intervals), i.e., neurons that showed simply an On (Fig. 2C).

The Off occurrence is usually based on an increased firing compared to baseline activity [22], [39]. For a better characterization in terms of separating between Off and Bl, we calculated the following measures, see Fig. 2B and 2C:

(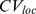) The coefficient of variation 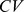 was computed by dividing the standard deviation of the inter-spike-interval (ISI) distribution by its mean. It is 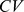  = 0 for regularly spiking neurons and 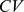  = 1 for irregular Poissonian spiking. To be relatively independent of slow variations in the firing rate we used a local version 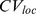, i.e., considering only two adjacent ISIs at a time [52].

(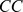) Typically the correlation coefficient characterizes the synchrony in neuronal firing (pairwise calculation): 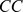  = 1 if two neurons fire synchronously, 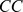  = 0 if firing is completely asynchronous. Since we consider several trials of one neuron (instead of synchronous trials of different neurons), 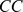 here characterizes the spike time precision from trial to trial (cf. [22]). The bin size was 0.05 ms for all pairs.

(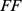) The Fano Factor is calculated from the population activity, i.e., the variance of the firing rate divided by its mean. 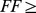
1 indicates unreliable neuronal firing, 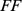  = 0 means reliable firing. The bin size was 0.125 ms. This analysis was done in Matlab.

### Robotic experiments

Tethered moths *A. ipsilon* were mounted on a Khepera III robot (K-Team, Vallorbe, Switzerland), see Fig. 1B. The insect body was immobilized inside a styrofoam block while the head was free in order to record the electroantennogram (EAG) [22], see Fig. 1, zoom 1. For electrical contact, the last 2–4 segments of one antenna were cut off and inserted into a glass pipette (Fig. 1, zoom 2) clamped by a micromanipulator and filled with (in mM) 6.4 KCl, 340 glucose, 10 Hepes, 12 MgCl_2_, 1 CaCl_2_, 12 NaCl. A silver wire inside the glass pipette served as recording electrode while another wire, the reference electrode, was inserted into the neck. The sensor was approximately 16 cm above the ground. An EAG acquisition board was embedded on the robot. The EAG signal was transmitted wireless via WIFI to a remote computer in order to be used as input for a MGC neuron model. This neuron simulation was performed in real-time (time steps  = 0.01 ms) using SIRENE, a C-based neural simulator (http://sirene.gforge.inria.fr). Neuron simulation, pheromone detection and robot control were performed in separate threads. A graphical user interface (written in Qt/C++) visualized both EAG input and neuron output. For more details see [22], [53].

Our cyborg (i.e., the robot using the antenna of a tethered moth as pheromone sensor) had to locate the pheromone source in an arena of 4 m length and 2.5 m width, see Fig. 1A. The whole set-up was placed in a Faraday cage (height 1 m) which was open to the upwind side. We assumed that the source was found whenever the cyborg entered a disk of 20 cm radius centered at the source. The cyborg always started at (x,y)  =  (0,0) m, the pheromone source was at (0,2) m. A fan was placed at (0,7) m providing a relatively constant wind in -y direction with an average velocity of 0.88
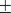
0.3 m/s (measured at the source location, 23 cm above the ground, i.e., the height of the center of rotation, with a hot wire anemometer Testo 425). The mean wind velocity was the same in all experiments and it was given to the robot as a fixed parameter. The airflow was rather turbulent than laminar. Additional wind velocity measurements one and two meters downwind from the source (on the centerline and on its left and right side) typically yielded a standard deviation between 20% and 30% of the corresponding mean values. We also estimated the Reynolds number to be 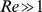, indicating turbulence. The source was a filter paper strip (approximately 5 cm long and 1.5 cm wide) with 10* µ*l of pheromone solution dropped on its tip, located approximately 16 cm above the ground. We used three different doses: minimum  = 0.1* µ*g/*µ*l, medium  = 0.3* µ*g/*µ*l and maximum  = 1* µ*g/*µ*l of main pheromone component (*(Z)*-7-dodecenyl acetate). The plume contour (indicated by black dashed lines in Fig. 4A, 4B and 5A) is defined as the parabolic region where 90% of all pheromone detections occurred. For trials that lasted longer than 3 min, the filter paper was replaced every trial, otherwise, we used one filter paper for two consecutive trials. The cyborg trajectories were recorded using path integration provided by the odometry tracking module of the Khepera III Toolbox (http://en.wikibooks.org/wiki/Category:Khepera_III_Toolbox).

We employed three reactive search strategies and one cognitive strategy, namely infotaxis [6]. Whenever the moth detected an odor patch the corresponding peak in the EAG activated the neuron simulator resulting in a multiphasic response as described above. The condition for a detection event in the simulated signal were three consecutive ISIs
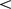
70 ms followed by an inhibitory phase (ISI
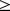
350 ms). This signal controlled the resulting robotic movements. We only exploited the On response and the inhibitory phase of the simulated neuronal signal. Onset and duration of all movement sequences subsequent to the surge were predefined or determined by a new detection event. The movements of our cyborg are clearly less precise than, for example, movements in computer simulations. This is due, for instance, to packet loss in the WIFI connection, odometry errors (i.e., imprecision in the path integration), and friction issues. All movement related data reported are thus to be considered approximate.

### Reactive search strategies

After each detection, the cyborg received a surge command leading to a straight upwind movement (approximately 5 cm). This was either followed by spiral casting (*sp* strategy) or by crosswind zigzagging (*za* and *ze* strategy) as illustrated in Fig. 2, right. The angle of the zigzag movement was 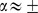
60° with respect to upwind direction, the step length was approximately 9 cm, doubling with each crossing of the centerline. If there were no more detections, zigzagging stopped after approximately 19 s, followed by spiral casting. The arithmetic spiral (*za*) was generated by 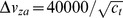, while the exponential spiral (*ze*) results from 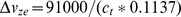, with 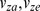 representing the speed differences between left and right wheel of the cyborg, and 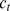 a time step counter. We did not explicitly compare arithmetic and exponential spiraling since the exponential spiral aimed at a further shortening of the trajectories including zigzag [22, Suppl. Information]. The cyborgs speed was approximately 5 cm/s.

### Cognitive search strategy

The cyborg was controlled by the infotaxis strategy [6]. In contrast to the continuous movements sequences detailed above infotaxis yields discrete steps as time is discretized (arbitrary units a.u.). At each time step the infotaxis program determined the next action which is either move one step of approximately 10 cm forward, backward, left, right or stay, depending on the information gathered by the agent, i.e., time and location of previous (non-)detections. The infotaxis algorithm chose the action that locally maximizes the expected information gain calculated as entropy reduction. Let 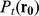 be the posterior probability distribution for the unknown source location 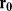:
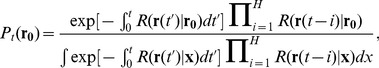
(1)where 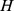 is the number of detections along the trajectory, and 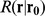 denotes the mean rate of detections at 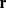 for a source at 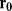. Given the odor plume model in [6] with an odor patch emission rate 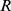, a lifetime 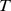 of the patches, a diffusivity 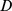, a wind 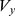, and an agent of size 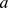 the mean detection rate can be calculated as:



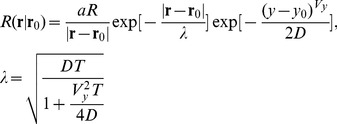
(2)The odor plume model entered our robotic experiments only in terms of predicting the expected rate of information acquisition but not for the actual detections. These were registered by the insect's antenna. The parameters were adjusted in a way that pure infotaxis simulations reproduced similar detection rates as measured for the reactive strategies: 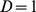, 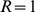, 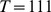 (a.u., chosen so that most patches survive until the end of each trial), 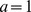, and 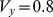 (see above). To account for higher stimulation doses, we increased 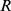 up to 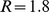. Since there was no characteristic difference in the resulting search trajectories compared to 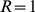 the data were pooled together. It has already been shown that the infotaxis algorithm is rather insensitive with respect to changes in these parameters [6], [30], [32]. The cyborg's speed was in between 2 cm/s and 6 cm/s (the speed changed smoothly because abrupt de- and acceleration caused noise in the EAG signal). The waiting time after each step was approximately 1 s and 3 s in case of a stay command.

## Analysis of the search trajectories

In total, we performed 428 successful trials using reactive search strategies and 85 successful trials using infotaxis (Table 1). Ideally, for reactive searching, the cyborg should not be able to locate the source when there is no pheromone. Owing to occasional false odor detections (Fig. 5D), the cyborg might nevertheless reach the source. Such reactive trials were considered as false-positives (Fig. 3A). However, in case of cognitive searching, such trials were considered to be successful as searching with infotaxis conceptually continues until the source is reached. The trajectories resulting from successful trials were analyzed and compared by computing the following quantities. We first calculated the success rates and the trajectory lengths. A trial was considered to be a failure when the cyborg left the experimental field. Because of some technical issues (see above), particularly occurring when using infotaxis, we additionally rejected all *it* trials longer than 125 time steps. Likewise, if the cyborg lost orientation for 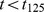 or if the source was reached in computer simulations but not in cyborg experiments (e.g., due to imprecisions, odometry problems) the corresponding trial did not enter the statistical analysis. The mean success rates obtained using reactive and cognitive searching are presented as simple bar plots in Fig. 3A and 3B, respectively. The trajectory lengths, as well as the horizontal deviation 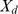, the number of turns, and the total number of detections are presented as notched box plots in Fig. 3C, 3D, 3F, 3G, 4E, 5B, and 5D. Thick horizontal bars indicate the median, box borders indicate the 2nd and 3rd quartile, notches represent the 95% confidence interval of the median, antennae indicate the range, and dots represent outliers (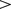
2.5 interquartile range). All statistical test (ANOVA, Pairwise Wilcoxon Rank Sum Tests) were performed in R (http://www.R-project.org/). Track-angle histograms (bin size  = 10°, percentage on the y-axis) show the distribution of track-angles, i.e., movement directions: 0 means straight upwind, 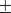
180° means moving downwind, 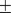
90° means a 90° crosswind orientation and so on. In order to estimate the horizontal deviation from the optimal path between start and source we calculated the horizontal deviation from x = 0, see Fig. 3E, 3F and 3G: 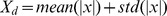 for each sample point (x,y) of a trajectory, then averaging over all trajectories of one type. We additionally computed the total number of turns, i.e. we counted all track-angles larger than 55° independent of the turning direction (Fig. 4E and 5B).
