# Correction: Reactive Searching and Infotaxis in Odor Source Localization

**DOI:** 10.1371/journal.pcbi.1004019

**Published:** 2014-11-17

**Authors:** The PLOS Computational Biology Staff

## Notice of Republication

This article was republished on October 28, 2014, to correct an error in displaying the PDF. Please download the PDF again to view it correctly. The previous version of the PDF is not available because an error prevented it from being viewed.
